# Stump appendicitis: a retrospective review of 3130 consecutive appendectomy cases

**DOI:** 10.1186/s13017-018-0182-5

**Published:** 2018-05-24

**Authors:** Enis Dikicier, Fatih Altintoprak, Kayhan Ozdemir, Kemal Gundogdu, Mustafa Yener Uzunoglu, Guner Cakmak, Feyyaz Onuray, Recai Capoglu

**Affiliations:** 1 Department of General Surgery, Istinye University Faculty of Medicine, Istanbul, Turkey; 2 Department of General Surgery, Sakarya University Research and Educational Hospital, Sakarya, Turkey

**Keywords:** Appendicitis, Stump appendicitis, Remnant appendicitis

## Abstract

**Background:**

Stump appendicitis is inflammation of remnant appendix tissue due to incomplete removal of the appendix. Due to appendectomy history, stump appendicitis diagnosis is usually delay and that can cause increase morbidity.

**Methods:**

Medical records of patients who had surgery for acute appendicitis at a single center from 2008 to 2017 were retrospectively reviewed. During the evaluation of medical records, patients that had a previous operation for acute appendicitis or had “stump appendicitis” as an exploratory finding in operation notes were included.

**Results:**

Appendectomy was performed in 3130 patients (2630 open surgeries and 380 laparoscopic surgeries). Stump appendicitis was diagnosed in five patients (0.15%). The appendectomies had been performed 4, 5, 7, 7, and 11 years previously. Mean time taken for surgery was 36 h after symptoms began. Open surgery was performed in three patients, laparoscopic procedures in others.

**Conclusion:**

Awareness of stump appendicitis before radiological examinations may facilitate accurate diagnosis and decrease the duration of the decision-making process, leading to decreased morbidity.

## Background

Appendectomy is currently the most common surgical operation worldwide [1]. Stump appendicitis is a rare condition beside the other common post-operative complications of appendectomy which are wound infection, bleeding, and intestinal obstruction.

Stump appendicitis is inflammation of remnant appendix tissue due to incomplete removal of the appendix [2]. This condition, which can be considered as recurring of acute appendicitis, may occur due to some technical and anatomical factors. Clinical findings are similar with acute appendicitis, as abdominal pain located on the right inferior quadrant is the most common sign [3]. This condition is not usually considered as preliminary diagnosis by clinicians at first referral due to previous appendectomy history. A delayed diagnosis may lead to delays in treatment and subsequently to an increase in morbidity.

In this retrospective study, four stump appendicitis cases out of 3130 consecutive appendectomies were evaluated and the medical histories of the subjects were reviewed.

## Methods

The charts of patients who had surgery for acute appendicitis at the General Surgery Clinics of Sakarya University Faculty of Medicine from 2008 to 2017 were retrospectively reviewed. During the evaluation of medical records, patients that had a previous operation for acute appendicitis or had “stump appendicitis” as an exploratory finding in operation notes were included. For these patients, we reviewed demographic data, time after the initial operation, symptoms at the time of referral, laboratory and radiological findings, and operation findings. Previous operation and pathology reports were reviewed, and previous appendectomy was confirmed.

## Results

Appendectomy was performed in 3130 patients with a diagnosis of acute appendicitis between January 2008 and November 2017 (2630 open surgeries and 380 laparoscopic surgeries). Perforated appendicitis in 621 (19.8%) patients, acute appendicitis in 2024 (64.6%) patients, and normal appendix (negative appendectomy) in 476 (%15.2) patients were found.

Stump appendicitis was diagnosed in five patients (0.15%). Three patients (60%) were male and two (40%) were female. The mean age was 32 (range 19–45). The appendectomies were performed 4, 5, 7, and 7 and 11 years previously using a conventional McBurney incision (open surgery). In the previous operation notes, the cases were recorded as perforated appendicitis in four patients (80%) and acute appendicitis in one patient (20%). In one of the patients who had been operated and confirmed due to perforated acute appendicitis, the localization of the appendix was recorded as retrocecal. The localization of the appendices of the other three patients was not recorded. The length of the appendices also was not recorded; the mean length of these appendices was 5.9 cm (4.9–7.2 cm) according to histopathologic reports.

The histopathological examination results were benign, and only inflammatory changes were observed in all patients.

One patient (20%) was referred to our clinic directly, and the remaining four patients (80%) were referred from other centers after an average of 2 days (range 1–3 days) of medical follow-up. During patient assessment at our clinic, three patients (60%) had classical physical examination findings consistent with acute appendicitis (tenderness and rebound in right lower abdominal quadrant) and the remaining two patients (40%) had acute abdomen (general tenderness, abdominal guarding, and rebound tenderness) and fever. An increase in the white blood cell (WBC) count was a common laboratory finding. The average WBC count was 14,300/mm^3^ (range 12,800–18,700/mm^3^).

**Fig. 1:**
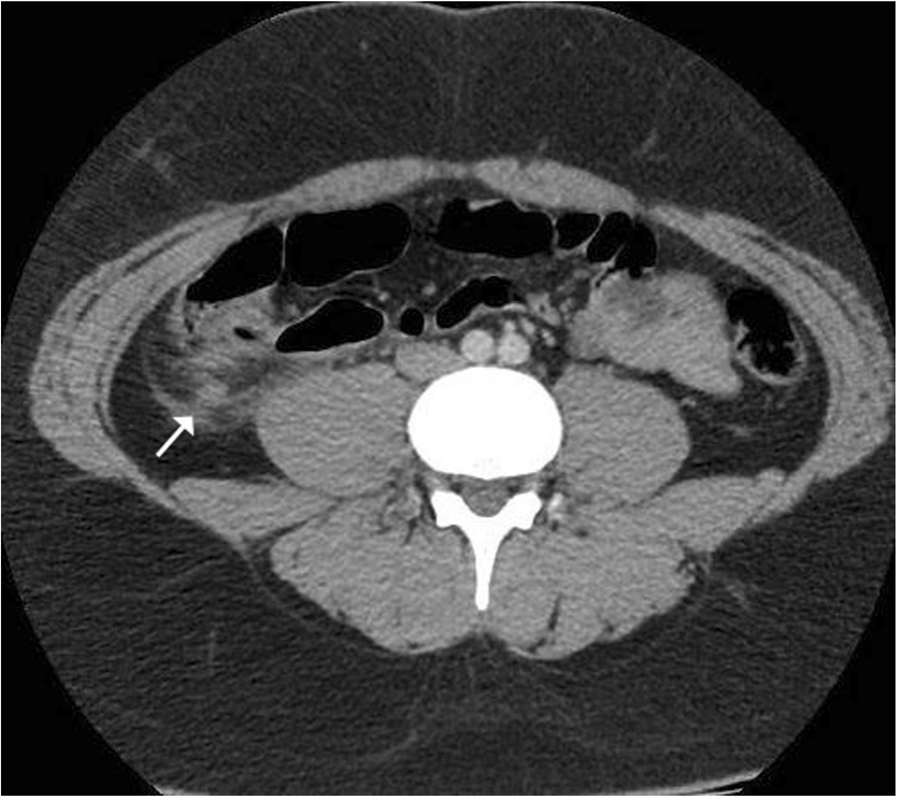
Abdominal CT scan. In the right lower quadrant, inflammation in the pericecal region and tip of the remnant appendix tissue (arrow) is observed

**Fig. 2:**
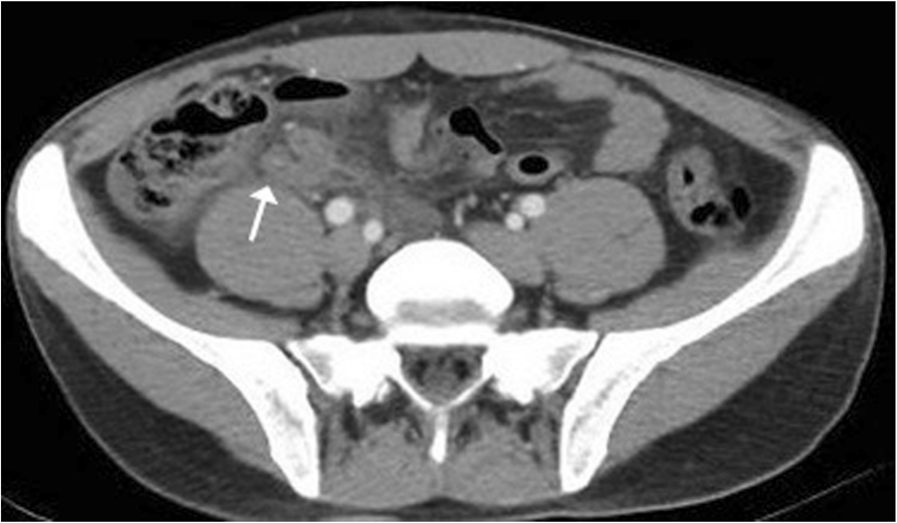
Abdominal CT scan. In the right lower quadrant, remnant appendix tissue in the pericecal region and fecalitis within the appendix tissue are observed

Conventional radiographic imaging results were normal in all patients. Abdominal ultrasonography (USG) exams revealed acute appendicitis in two patients (40%). In the remaining three patients (60%), USG revealed inflammatory changes around the pericecal region. In these patients, the appendix was not observed; therefore, abdominal computerized tomography (CT) was performed, which revealed inflammation around the pericecal region, localized abscess formation, and thickening of the remnant appendix wall (Figs. 1 and 2).

On average, patients had surgery 36 h (range 24–72) after symptoms began. During surgery, one patient (20%) was under spinal anesthesia, and the remaining four patients (80%) were under general anesthesia. In three patients (60%), the surgeries were held as open operations using a McBurney incision line. In the other two patients (40%), diagnostic laparoscopy was performed. In the two diagnostic laparoscopy patients, one procedure (50%) was converted to open surgery due to dense attachments at the appendectomy lodge. In the other patient, operation was completed as laparoscopic. Perforation did not occur in any of the patients. Localized abscess formation was observed in three patients, and only inflammatory changes were observed in the other two patients.

**Fig. 3:**
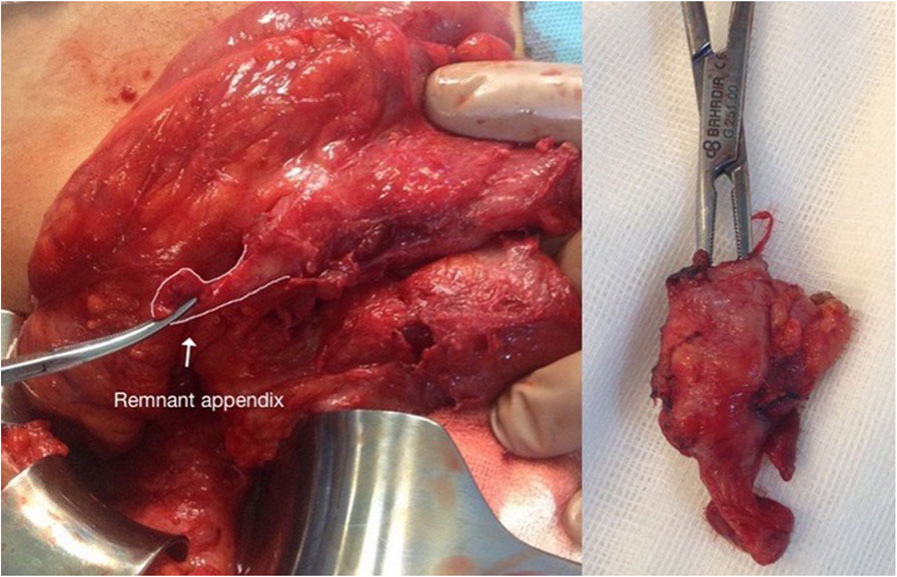
Intraoperative view of stump appendicitis due to remnant appendix tissue

In three patients (60%), the location of the remnant appendix was subserous, and in two patients (40%), there was still a residual remnant appendix tissue (Fig. 3) although the location was not subserous. No remnant meso-appendix was detected within remnant appendix tissues. Remnant tissue was isolated through the entrance part on the cecum and ligated with 2/0 absorbable suture on that localization. The mean length of the remnant appendix was 2.7 cm (range 1.8–4.2). In all patients, the remnant appendix tissue was easily excised; the appendix radix was tied with a double tie using 2/0 Vicryl and was not inverted into the cecum. The post-op period was normal, and patients were discharged from the hospital within 2 days on average (range 1–4). Histopathological exams revealed acute stump appendicitis in all cases.

## Discussion

Appendectomy is currently the most common surgical operation worldwide [2]. After appendectomy, various morbidities including wound infection may be encountered. Remnant appendix tissue inflammation after appendectomy is called stump appendicitis, a very rare condition. The lifelong probability of developing acute appendicitis is estimated at 7%, but the probability of developing stump appendicitis is much lower (1/50,000) [3]. In our study, this rate was very low (0.15%).

As expected, the symptoms and signs of stump appendicitis are the same as for classical acute appendicitis [4]. As such, clinicians usually do not suspect or, worse, disregard a probable diagnosis of stump appendicitis in patients with a history of appendectomy who present with clinical signs of acute appendicitis. This may lead to a delay in diagnosis, which may lead to future complications. Kumar et al. [5] reported suppurative appendicitis during laparoscopic exploration in a patient with a previous history of appendectomy and abdominal pain at the time of referral. Manoglu et al. [6] described a patient with cecal necrosis secondary to stump appendicitis who was referred to the hospital two times with complaints of abdominal pain. In our study, the average time to surgery was 2 days after referral of the patient. This is consistent with previous findings that stump appendicitis is difficult to diagnose. In three of our cases (60%), abscesses were detected during surgery; however, more complicated conditions were not encountered.

In a literature review conducted by Kanona et al. [7], the time it took for stump appendicitis to develop in patients with inadequate appendectomy ranged from 9 weeks to 50 years. In addition, Onder et al. [8] conducted two case studies and found that a history of appendectomy dated back to 4 months in one patient and 4 years in the other patient. In our study, the shortest time from first appendectomy to the development of stump appendicitis was 4 years; however, a much shorter time period is possible.

Establishing a diagnosis of stump appendicitis is difficult, and delayed diagnosis is established only after a certain period of clinical follow-up [5]. Even in the patient whose first referral was to our clinic, the patient underwent surgery 24 h after referral because, although a radiological exam done in the initial referral suggested stump appendicitis, a second radiological exam and second opinion were deemed necessary to confirm the diagnosis. This observation suggests that a delay in surgical decision in cases of stump appendicitis is due not only to the difficulty of establishing a diagnosis during the first referral, but also to the desire of the surgeons to feel confident about the diagnosis.

Clinical signs and laboratory findings of stump appendicitis are similar to those of primary acute appendicitis; therefore, radiological methods are more useful for differential diagnosis. In USG, remnant appendix tissue can be detected as a tube extending from the right iliac fossa or retrocecal region to the cecum [9, 10]. In abdominal CT, inflammation in the pericecal region, abscesses, thickening of the cecum and terminal ileum, and free-floating fluid at the pericecal and paracolic region are the likely findings [11–13]. In some cases, the appendix stump may be inflamed and edematous, and in other cases, fecalitis may occur [14]. In all of our cases, preoperative radiological exam findings revealed remnant appendix tissue and inflammation.

According to the literature, remnant appendix tissue > 5 mm in length is a risk factor for fecalitis and stump appendicitis [15]. The length of the remnant appendix ranges from 0.5 to 6.5 cm in patients who have undergone surgery with a diagnosis of stump appendicitis [8, 12]. In our study, the mean length of the remnant appendix was 2.3 cm. In addition to the lack of experience of the surgeon, reasons for longer remnant tissue include a subserous or retrocecal position of appendix tissue and inadequate dissection during laparoscopic appendectomy [16, 17]. A retrocecal position of remnant appendix tissue was found in three out of four patients in our study. This suggests the anatomic location of the appendix is a significant factor in the development of stump appendicitis.

There are some comments in the literature that appendectomy is performed by incomplete exposure of the radix of the appendix, so excess residual part is left and stump appendicitis takes place after laparoscopic appendectomy [1]. However, most of the stump appendicitis in the literature are reported after open appendectomies. [3]. The important thing is performing the appendectomy after complete exposure of the meso-appendix, taenia coli of the cecum, and appendico-cecal junction and ligation of recurrent or accessory branch of the appendiceal artery (artery of Seshachalam), independent from the choice of method: laparoscopic or open [18, 19].

Histopathologic examination revealed no remnant meso-appendeceal tissue. Perforation was present in the four of the stump appendicitis cases. The inflammation may be a risk factor for stump appendicitis. We suggest that intensive inflammation during appendectomy may prevent to recognize the subserosal localization of appendix or makes it difficult to isolate the appendix through the cecal entrance area; each of the situations may increase the risk of stump appendicitis. The rate of stump appendicitis after perforating appendicitis in our study (4/621, 0.64%) is similar with the rates reported in the literature. Remnant tissue was sub-serosal in the three of these four cases. These findings support our suggestion.

There is no standardized surgical approach for probable stump appendicitis, but it has been reported that laparoscopy is superior to open surgery because it provides a better viewing angle, which leads to a better differential diagnosis [20].

## Conclusion

Delays in the diagnosis of stump appendicitis continue to be an issue. Awareness of stump appendicitis before radiological examinations may facilitate accurate diagnosis and decrease the duration of the decision-making process, leading to decreased morbidity.
